# Muscle and Adipose Tissue Communicate with Extracellular Vesicles

**DOI:** 10.3390/ijms23137052

**Published:** 2022-06-24

**Authors:** Sophie Rome

**Affiliations:** CarMeN Laboratory, INSERM 1060/INRAE 1397, Lyon-Sud Faculty of Medicine, LYON 1 University, 69301 Pierre Bénite, France; srome@univ-lyon1.fr

**Keywords:** adipose tissue, muscle, extracellular vesicles, exosomes, microparticles, microvesicles, stem cells, adipocytes

## Abstract

In numerous body locations, muscle and adipose tissue are in close contact. Both tissues are endocrine organs that release cytokines, playing a crutial role in the control of tissue homeostasis in health and diseases. Within this context, the identification of the signals involved in muscle–fat crosstalk has been a hot topic over the last 15 years. Recently, it has been discovered that adipose tissue and muscles can release information embedded in lipid-derived nanovesicles called ‘extracellular vesicles’ (EVs), which can modulate the phenotype and the homeostasis of neighboring recipient cells. This article reviews knowledge on EVs and their involvement in the communication between adipose tissue and muscle in several body locations. Even if the works are scarce, they have revolutionized our vision in the field of metabolic and cardiovascular diseases.

## 1. Introduction

It is now well established that throughout the body, muscle and adipose tissue interact, and the identification of the signals involved in muscle–fat crosstalk has been a hot topic over the last 15 years. This has led to the discovery that these tissues are endocrine organs, which release myokines (cytokines released from skeletal muscle in response to physical activity) and adipokines (cytokines constitutively released from adipose tissue). These cytokines play a crucial role in the control of both tissues homeostasis in health and diseases [1,2,3].

Muscles and adipose tissue present in different anatomical locations have different structural and functional properties. Muscle is a tissue made up of fibers capable of contracting to produce movement and to participate in various functions essential to the life of the organism (breathing, digestion, etc.) and its adaptation to the surrounding environment (locomotion, thermoregulation, etc.); i.e., skeletal muscles work to produce force for maintaining the posture and locomotion and to regulate whole-body glucose homeostasis; cardiac muscle is involved in the contraction of the heart; and smooth muscles constitute much of the musculature of internal organs (all blood vessels except the smallest, intestines, the uterus, etc.). Adipose tissue, fat mass or body fat, is a connective tissue containing fat cells. These cells are separated by an extracellular matrix and are called “adipocytes”. Three types of white, beige, and brown adipocytes constitute the white adipose tissue (WAT), mixed adipose tissue (white/beige), and brown adipose tissue (BAT). White adipocytes store energy provided by food in the form of triglycerides. Brown and beige adipocytes store and then mobilize triglycerides to produce heat. Variability in the proportion of brown/beige adipocytes and white adipocytes may contribute to variability in energy expenditure. The development of muscles and adipose tissue depends on complex processes that are both continuous and inter-connected. During embryogenesis, embryonic stems commit to particular differentiation pathways (myoblasts or pre-adipocytes); then they increase both in number (hyperplasia) or fuse (i.e., muscle syncytium), and in volume (hypertrophy) during the phases of postembryonic growth. Knowledge about tissue growth suggests prioritization or a competition for the growth of muscles compared to that of adipose tissue. Indeed, the growth of muscle precedes that of adipose tissue, and growth allometric analysis shows that the growth speed of adipose tissue increases with age, while that of muscle decreases [4]. The relative proportion of these different tissues is regulated throughout life, and the alteration of this ratio, either during the prenatal stage or after birth, impairs the control of body weight and fasting glycemia, and it can contribute to diabetes and cardiovascular diseases.

Recently, it was discovered that cells from tissues, including adipose tissue [5,6] and muscles [7], can release information embedded in lipid-derived nanovesicles, which can modulate the phenotype and the homeostasis of recipient cells [8]. This discovery has highlighted a cytokine-independent pathway by which muscle and adipose tissue exchange information to control the respective mass of each tissue. In addition, other studies have demonstrated that extracellular vesicles (EVs) participate in the development of metabolic diseases [9]; therefore, understanding the message conveyed by these EVs is very important and is the subject of more and more work. This article reviews knowledge on EVs and their involvement in the communication between adipose and muscle tissues in several body locations. Even if data in the literature are scarce, they have revolutionized our vision on how muscles and adipose tissue talk to each other in the field of metabolism and endocrinology.

## 2. The Different Types of Extracellular Vesicles

**Figure 1:**
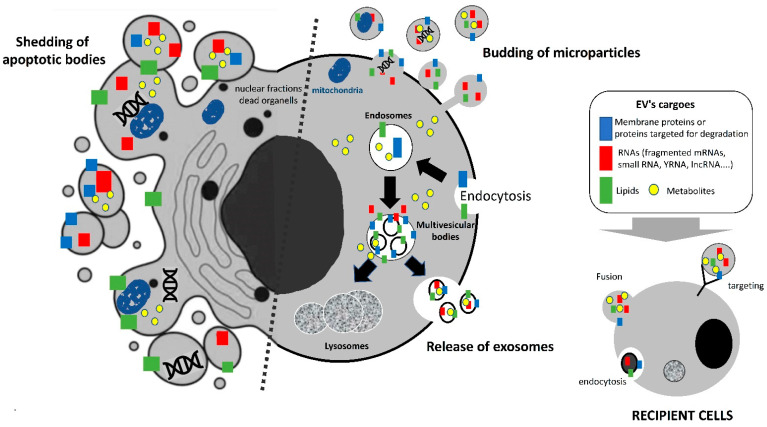
Different populations of extracellular vesicles released from almost all cell types. The relative proportion of microvesicles and exosomes is variable and depends on each cell type. Apoptotic bodies contain information from dying cells, such as DNA, fragmented RNA, and organelle material. Mitochondrial DNA has been described in muscle- and adipocyte-derived EVs. Exosomes and microparticles are released by live cells and can target recipient cells either via receptor interactions, direct fusion, or endocytosis/pinocytosis.

Extracellular vesicles (EVs) are small, lipid-derived nanovesicles released from a great variety of cells [10]. According to their mode of biogenesis, their composition and their size, three main subgroups of EVs are recognized: exosomes (EXO); microparticles (MP), or ectosomes; and apoptotic bodies (AP) (Figure 1). It has been noted that muscle and adipose tissue likely release the three types of vesicles, and we have already well described their characteristics in previous reviews [5,6,7]. Among these EVs, EXOs are nanosized (50–100 nm) vesicles formed during the inward budding of the limiting membrane of the late endosomes (a subpopulation of endosomes enrich in BMP). This microautophagy leads to the generation of multivesicular bodies containing intraluminal vesicles (ILVs). MVBs can either fuse with lysosomes to degrade their cargos, or they can migrate along the microtubules, a dynamic process regulated by cholesterol [11], to release their content outside the cell by fusing with the plasma membrane. In the microenvironment, the released ILVs are called ‘exosomes’. The molecular mechanisms that regulate the fate of MVBs are not completely understood and likely depend on intracellular homeostasis; i.e., starving will favor the process of ILV degradation for nutrient recycling, and the release of exosomes will be decreased. On the other hand, the generation of toxic components in response to external stimulus (including transfected siRNAs [12]) will be exported within EXOs [13]. EXOs are rich in cholesterol and in desaturated molecular species of phospholipids [14]. Proteins contained in EXOs are involved in their biogenesis (protein from the ESCRT complex [15] or tetraspanins [16]), are proteins targeted for degradation [17], or are signaling proteins [18]. Adipocyte- and muscle cell-derived EXOs have specific protein compositions, i.e., muscle-EXOs are enriched in proteins for neuromuscular development and cell differentiation, and adipocyte-EXOs are rich in the proteins involved in RNA processing and translation and from the extracellular matrix (ECM) [6]. EXOs also contain noncoding RNAs, i.e., long noncoding RNA, microRNAs (miRNAs), piwi protein-interacting RNA (piRNA), small nuclear RNA (snRNA), small nucleolar RNA (snoRNA), small Cajal body-specific RNA (scaRNA), circular RNA (circRNA), sc RNA Y, natural antisense RNA (asRNA), ribosomal RNA (rRNA), and vault RNA (vRNA) [19]. EXOs are enriched in small RNA species (<200 nucleotides), including short transcripts or fragmented mRNAs [20] compared to the cellular RNAs, which are rich in full-length long RNA species. In the case of the miRNAs contained within skeletal-muscle-cell-derived EXOs, their concentration in EXOs correlates with their concentration in the cells, for the majority of them [21]. However, for some miRNAs, the presence of specific motifs have been found to be enriched in those exported into EVs and to be involved in their export [21,22], suggesting that specific and unspecific mechanisms cooccur in muscle cells for the export of miRNA. This has not been studied for the miRNA contained in adipose-tissue derived EVs. In addition to RNA, the presence of mitochondrial DNA has been found in EVs released in vitro from differentiated murine myotubes [23] and from adipocytes [24].

The two other types of extracellular vesicles (MPs and APs) have larger sizes than EXOs. Both APs and MPs contain plasma membrane-associated proteins and lipids, and RNAs from the cytosol. APs also contain DNA. APs (500 nm-5μm) are generated from the disassembly of apoptotic cells. They contain nanoliters of the fragmented cells, including mRNAs, lnRNAs, small RNAs, lipids, plasma membrane proteins, and possibly DNA [25]. On the other hand, MP (100–400 nm) are formed during the repair of small lesions in plasma membranes in response to a rapid influx of Ca2+. The loss of plasma membrane, which is asymmetric in phospholipid distribution, regulates membrane flippase, floppase, and scramblase activities, leading to phosphatidylserine (PS) and phosphatidylethanolamine (PE) exposition on the outer membrane leaflet, and the activation of contractile proteins involved in their release [26]. As PS is a negatively charged phospholipid, it confers to MP a higher clotting capacity than EXOs [27]. Once released into the intercellular space, all EVs are either degraded or cleared by macrophages, or they can integrate into neighboring cells through receptor-mediated endocytosis, macropinocytosis, or membrane fusion (Figure 1). It has been demonstrated that EVs have virus-like properties and can transfer their cargos to modulate the recipient cells’ phenotypes and their ultimate fates [8]. If we consider the diversity of the material contained in EVs, it appears that EV content can interact at different levels in the recipient cell and can send a much more complex signal than the one associated with soluble proteins. Indeed, in addition to their lipid and protein content, the three types of EVs also differ in their RNA content [28]. Depending on their mode of integration into the recipient cells, i.e., fusion or endocytosis, EV cargos will be released either into cytoplasm or MVB/lysosomes and, thus, into different subcellular compartments. We have recently found that many miRNAs contained in skeletal muscle-released EVs have sequences for nuclear import, also indicating an epigenetic action in EXO-miRNA in target cells [21].

**Figure 2:**
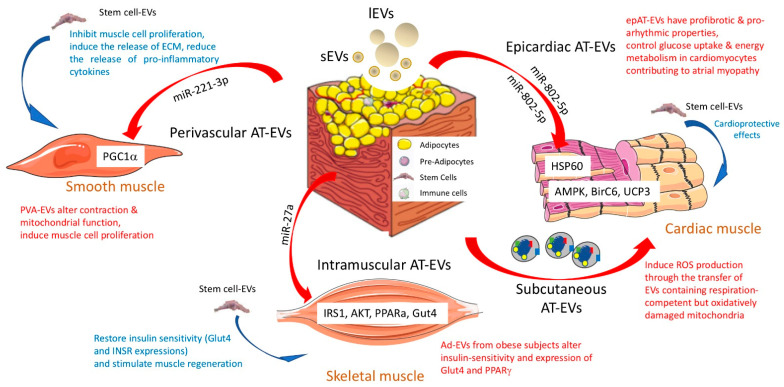
Biological functions of adipose tissue-derived extracellular vesicles on the three different muscle cells (cardiac muscle, smooth muscle, and skeletal muscle) in pathological contexts (red). In blue, the specific functions of stem cell-EVs are highlighted. For each type of adipose tissue, the role of miRNAs contained in EVs is indicated, and their target genes are mentioned in the muscle tissue. AT = adipose tissue; EVs = extracellular vesicles; ECM = extracellular matrix; sEV = small EVs with exosome-like properties; lEV = large EVs from the budding of the plasma membrane [29].

**Figure 3:**
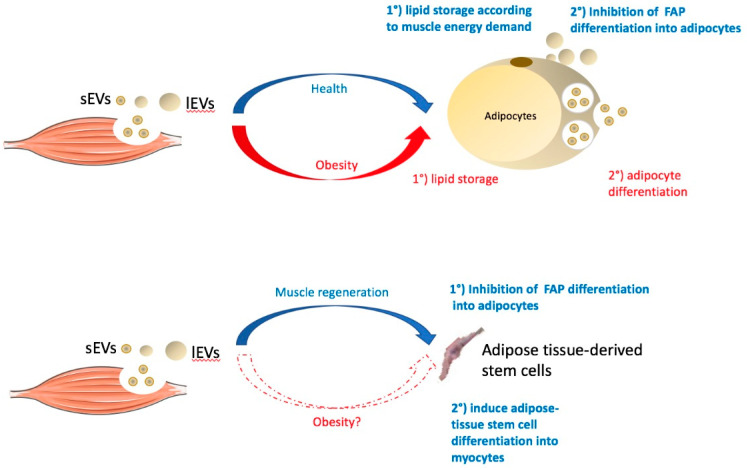
The role of extracellular vesicles released from skeletal muscles on adipocytes or on adipose tissue stem cells in healthy (blue) or obese (red) situations (see below). FAP = fibro-adipogenic progenitor cells; sEVs = small EVs with exosome-like properties; lEVs = large EVs budding from the plasma membrane.

Given the overlapping sizes and compositions between EXOs, MPs, and APs, it is very difficult to obtain pure populations for each EV subtype. Therefore, the MISEV2018 guidelines recommend using the term of small vesicles (sEVs) for <200 μm nanovesicles with exosome-like characteristics, and large vesicles (lEVs) for >200 μm nanovesicles pelleted at low speed during EV purification [29] (Figure 2 and Figure 3). In agreement, and in order to take into account this bias, we will not describe the origin of the vesicles studied in each article mentioned below, but we invite readers to take a good look at the material and method of each article in order to understand the origin of these EVs and to compare what is comparable in terms of biological activities.

## 3. Biological Effects of Adipose Tissue-Released EVs on Muscle Cell Homeostasis

Adipose tissue (AT) is loose connective tissue composed of stem cells, preadipocytes, macrophages, neutrophils, lymphocytes, and microvascular endothelial cells, which, together, are referred to as the stromal vascular fraction. It has been demonstrated that all of these cell types release EVs [6], which, individually, can contribute to the regulation of muscle cell homeostasis (Figure 2).

### 3.1. Adipose Tissue and Cardiac Cells

#### 3.1.1. Epicardiac Adipocyte-Derived EVs

Ischemic heart disease and endomyocardial fibrosis are the primary causes of heart failure, and it is well established that perivascular and epicardiac AT participates in cardiac cell alteration during metabolic diseases through the release of proinflammatory cytokines [30]. In the context of atrial fibrillation, it has been found that epicardiac adipose tissue also exports EVs (epAT-EVs) with profibrotic and proarrhythmic properties [31]. EpAT-EVs contain proteins involved in fibrosis, angiogenesis, and coagulation. In vivo, these profibrotic and proarrhythmic properties have been found to be increased when epAT-EVs are isolated from patients suffering from atrial fibrillation (AF) compared to patients without AF (10 ug injected into rat left ventricular anterior). This result was correlated with the fact that EpAT-EVs from AF patients carry higher amounts of proinflammatory and fibrotic cytokines and fewer amounts of anti-inflammatory cytokines. In addition, the concentrations of miRNAs involved in fibrosis and collagen synthesis (i.e., miR-146b, miR-133a, and 29a) are altered in epAT-EVs isolated from patients with AF vs. epAT-EVs from patients without AF. These data demonstrate that epAT-EVs can regulate myocardium homeostasis and, for the first time, that epAT-EVs participates in the development of atrial myopathy. In that context, it would be interesting to determine whether some miRNAs, enriched in the atria of patients suffering from AF vs. non-AF patients, could be transferred from the epicardiac AT though epAT-EVs [32].

In the context of obesity, it is known that insulin resistance in cardiac myocytes, associated with the ectopic growth of adipose tissue within and around the heart, contributes to diabetic cardiomyopathy and, subsequently, heart failure. In vitro data suggested the role of adipocyte-derived EVs on the alteration of cardiac cell glucose homeostasis in [33]. In that study, hypertrophic adipocyte-released EVs (Ad-EVs) altered insulin-signaling and glucose uptake in neonatal rat ventricular myocytes. This result was correlated with an increased amount of miR-802-5p in Ad-EVs from hypertrophic adipocytes that target HSP60 in recipient cardiomyocytes. As a consequence of HSP60 deficiency, the cardiomyocytes had increased levels of unfolded protein response (UPR) and reactive oxygen species (ROS), both of which contribute to insulin resistance associated with mitochondrial dysfunction [33]. The importance of EpAT-EVs from hypertrophic adipocytes on the development of diabetic cardiomyopathy was also demonstrated in a model of high-fat diet obese mice (DIO) in [34]. In this study, intravenous injections of obese EpAT-EVs (1-5×10^8^ EVs) exacerbated myocardial ischemia/reperfusion injuries. Combining elegant in vitro and in vivo experiments, the authors identified miR-130b-3p as the EpAT-EVs....which mediated cardiomyocyte apoptosis. The proapoptotic effect of EpAT-EV miR-130b-3p correlated with its binding to the 3’UTR regions of AMPKα1/α2, Birc6, and Ucp3 in recipient cardiomyocytes [34]. This study demonstrated that, in the context of obesity, the antiapoptotic and cardioprotective effects of EpAT-EVs are lost and contribute to myocardial dysfunction.

Beside this ‘short distance’ message between epicardial AT and cardiac cells, a ‘long distance’ message between subcutaneous AT and cardiac cells was also suggested in [24]. First, the author demonstrated that adipocyte-derived EVs from in vitro adipocytes are taken up by cardiomyocytes. Second, palmitate-treated adipocytes released more EVs, than the control cells, and these EVs contained respiration-competent but oxidatively damaged mitochondria. The intravenous injection of 3 μg of these palmitate-treated adipocyte-derived EVs stimulated ROS production and caused mitochondrial dysfunction in cardiac tissue in vivo. This effect was transient and disappeared 2 h post-injection, demonstrating that an adaptive mechanism in cardiomyocytes restores energetic and redox homeostasis. Third, obese, metabolically unhealthy individuals have more circulating EVs that carry more mitochondrial and oxidized proteins compared to lean individuals. The authors of the study proposed the concept that the induction of ROS by AT-EVs (also observed when cardiomyocytes are treated with ‘obese’ EpAT-EVs [32,33]) could offer a kind of protection to cardiomyocytes against acute oxidative stress during lipotoxic or ischemic stresses induced by obesity [24].

#### 3.1.2. Adipose Tissue Mesenchymal Stem Cell-Derived EVs

Inside adipose tissue, adipose-derived mesenchymal stem cells have emerged as important actors for cardio-protection because of their capacities to stimulate angiogenesis and to inhibit apoptosis [35]. Indeed, AT-derived stem cells can be easily isolated and have a strong capacity to proliferate and to differentiate into both cardiomyocytes and endothelial cells. It has been demonstrated that AT-derived stem cells release EVs (AT-SC-EVs) with cardioprotective effects: i.e., AT-SC-EVs isolated from the inguinal fat pad have a protective effect on the myocardium against ischemia/reperfusion injuries [36]. Rats subjected to ischemia/reperfusion injury and treated with 400 ug of AT-SC-EVs at the beginning of the reperfusion had attenuated apoptosis, higher cardiac cell viabilities, and reduced necrosis compared to untreated animals in [36]. This result was explained by the stimulation of Wnt/beta-catenin signaling pathways by AT-SC-EVs in the recipient cardiac cells. Based on this result, an ‘EVs’ therapy can be envisaged to protect the heart from ischemia. An interesting preclinical study associated the beneficial effects of AT-SC-EVs on cardiac cell protection with their loading of proangiogenic miRNA (i.e., miR-126) [37]. As a result, miR-126-AT-SC-EVs promoted myocardial repair after ischemia, as expected, but also promoted angiogenesis and significantly decreased inflammation [37].

Similarly, it was demonstrated that AT-SC-EVs have a benefic effect against damage induced by myocardial infection in [38]. In a model of myocardial infection induced by left anterior descending coronary artery ligation, the injection of 2.5 10^12^ AT-SC-Evs in the inferior vena cava 1 h post-ligation reduced collagen fiber accumulation and reversed myocardial fibrosis and cardiac cell apoptosis. In vitro, AT-SC-Evs reversed hypoxia-induced apoptosis in cardiac cells by activating the S1P/SK1/S1PR1 signaling pathway. Interestingly, the same pathway was regulated in macrophages treated with AT-SC-Evs, leading to the polarization of macrophages into anti-inflammatory M2, which attenuated myocardial fibrosis [38]. These data demonstrate that AT-SC-EVs are important paracrine signals that can participate in cardiac muscle homeostasis. It is presently not known whether obesity alters these EV benefic properties. In addition, as stem cells from epicardiac and ventricular adipose tissue are different in terms of their proliferative and survival capacities, it would be interesting to determine whether their respective released EVs also have specific properties in cardiac muscle [39].

### 3.2. Adipose Tissue and Smooth Muscle Cells

#### 3.2.1. Perivascular Adipocyte-Derived EVs

Smooth muscle cells, which are cover the walls of all internal organs and blood vessels, are in close contact with adipose tissue. Recently, it was demonstrated that mesenteric adipose tissue (MAT) releases EVs originating from perivascular adipocytes (PVA-EVs), which can be incorporated into recipient smooth muscle cells [40]. Compared to the releasing perivascular adipocytes, PVA-EVs are enriched in small RNAs, including miRNAs. A similar result was found for EVs released from skeletal muscle cells, which are also enriched in shorter RNA species (≤200 nucleotides) compared to their releasing muscle cells, in [7], underlying a generic role for EV-small-RNAs in intercellular communication between cells. After 16wk of a high-fat diet, mice had larger adipocytes in MAT and altered expressions of the genes involved in the contraction of arterial smooth muscle cells. MATs in obese animals were found to release more EVs enriched in miR-221-3p in [40]. The authors demonstrated that miR-221-3p participated in the proliferative and pro-migrating effects of PVA-EVs on smooth muscle cells through their binding on PGC1α mRNA 3’UTR. As a result, the recipient smooth muscle cells had mitochondrial dysfunction. In vivo, lean mice treated with PVA-EVs from obese mice (30 ug/intraperitoneal injection) had a decreased expression of proteins involved in contraction and greater adventitia-to-media ratios. A similar phenotype was found when the animals were treated with miRNA-221-3p only, validating the role of this miRNA in the biological effect of ‘obese’ PVA-EVs. Taken altogether, these data demonstrate an EV-mediated, PVA-to-smooth-muscle-cell signaling axis, regulating smooth muscle cell homeostasis, which is altered in a context of obesity-induced diabetes [40].

#### 3.2.2. Adipose Tissue Mesenchymal Stem Cells-Derived EVs

Conversely to adipocyte-derived EVs, adipose mesenchymal stem cell-derived EVs (AT-MSC-EVs) have an inhibitory effect on the proliferation and migration of smooth muscle cells through their actions on the phosphorylation of MAKP and AKT in these recipient cells [41]. In addition, AT-MSC-EVs modulate the secretome of smooth muscle cells, which express less Il-6 and MCP-1 involved in immune responses and cell migration. In agreement, one study found that the intraperitoneal injection of AT-MSC-EVs decreases the neointimal thickness in a mouse model of vein-graft bypass, providing a proof-of-concept that AT-MSC-EVs can be used in the treatment of neointimal hyperplasia [41]. Furthermore, the positive effect of AT-MSC-EVs on the release of extracellular matrix proteins (deposition of fibrillar collagen and elastin) by smooth muscle cells was demonstrated in vitro by using a model of 3D culture system in [42], suggesting that AT-MSC-EVs could be used for vascular grafting applications. Taken altogether, these data indicate that AT-MSC-EVs can modulate the microenvironment of smooth muscle cells (i.e., modulation of cytokine and extracellular matrix release) and, thus, indirectly impact communication between smooth muscle cells and immune cells.

### 3.3. Adipose Tissue and Skeletal Muscle Cells

#### 3.3.1. Adipocyte-Derived EVs

Recent data suggest that Ad-EVs might participate in the alteration of whole-body glucose metabolism through their deleterious actions on SkM insulin-induced glucose uptake in the context of obesity [43]. It has been demonstrated that EVs released from palmitate-treated 3T3 adipocytes, in order to mimic an obese state, are incorporated at a higher rate in SkM cells than when originating from untreated adipocytes. Interestingly, lipidomic analyses of adipose tissue-derived EVs from high-fat diet obese mice have revealed a strong enrichment in palmitate vs. those isolated from adipose tissue-derived EVs from standard diet mice [44]. Considering all these data, it appears that the lipid composition of EVs is an important parameter for the incorporation of EVs into target cells. A too rich diet in palmitate, associated with the development of metabolic diseases, could participate in modifying the composition of EVs and, therefore, modify the crosstalk between adipose and muscle tissues, and vice versa [45]. In addition, Ad-EVs released from the adipose tissue of high-fat diet obese mice have been found to be enriched in miR-27a, and this was correlated with an increase in the plasma of obese animals (also observed in the plasma from obese children) [43]. In vitro, the incorporation of miR-27a-enriched Ad-EVs in recipient SkM cells reduces the insulin-stimulated phosphorylation of IR-1 and AKT and reduces the mRNA levels of PPARα, IRS-1, and GLUT4 in muscle cells. Conversely, a study on the effects of Ad-EVs from omental and subcutaneous adipose tissue from obese subjects did not confirm that obese Ad-EVs alter SkM insulin sensitivity [46]. Of note, in this last study, the authors only quantified the insulin-response after Ad-EV treatment, but not at basal state without insulin. Thus, it is not known whether the ratio p-AKT/AKT, representing insulin-sensitivity, is altered, as in [45].

#### 3.3.2. Adipose Tissue Mesenchymal Stem Cell-Derived EVs

In a recent report, it was found that the infusion of adipose tissue mesenchymal stem cells (AT-MSC) restored GLUT4 and INSR expressions in the SkM of diabetic rats involved in glucose uptake in response to insulin [47]. A parallel study demonstrated that the infusion of AT-MSC in high-fat-diet obese mice lead to an amelioration of their glucose tolerance, demonstrating that AT-MSC secrets specific proteins and factors able to control SkM glucose uptake [48]. Among these secreted factors, the role of AT-MSC-EVs in the crosstalk between AT-MSC and SkM was demonstrated by using a model of cardiotoxin-induced SkM injury [49]. Thirty minutes before muscle injury with cardiotoxin I, mice received 100 μL AT-MSC-EVs released in vitro from 1 × 10^6^ human subcutaneous MSC through intravenous injections. Five days later, these mice had large, newly formed fibers and decreased numbers of differentiating satellite cells (Pax7^−^/MyoD^+^) compared to PBS-treated animals, demonstrating that AT-MSC-EVs improves muscle regeneration. Interestingly, both the whole secretome and the AT-MSC-EVs of AT-MSC decreased the number of Pax7^+^/MyoD^+^ cells, but the decrease in Pax7^−^/MyoD^+^ cells was specific to AT-MSC-EVs [49]. The use of AT-MSC-EVs to stimulate muscle regeneration was also tested in a mouse model of hindlimb ischemia in [50]. Immediately after the intervention, the injection of AT-MSC-EVs, successively, intravenously, and intramuscularly, protected muscle tissue against ischemia-induced damage compared to the saline group. The muscle had higher expression levels of MyoD, Myf5, and Pax7, which are involved in myoblast proliferation and differentiation [50]. Taken altogether, these data demonstrate that AT-MSC-EVs participate in maintaining muscle mass. It is presently not known whether obesity affects the composition and biological actions of AT-MSC-EVs on muscle regeneration.

#### 3.3.3. Adipose Tissue Macrophage-Derived EVs

In a context of obesity, the accumulation of proinflammatory macrophages in adipose tissue has an important role in the development of systemic inflammation, which is now recognized as a major actor in the development of insulin resistance in insulin-sensitive tissues, including SkM [51]. A number of studies have demonstrated that proinflammatory cytokines, secreted from tissue macrophages, directly inhibit insulin-sensitivity in SkM [52]. Recently, a study demonstrated that adipose tissue macrophage-derived EVs (AT-MEVs) participate in the development of SkM insulin resistance in a model of high-fat-induced obese in mice [53]. In this study, visceral AT-MEVs from normal or obese mice were isolated and used to treat SkM cells. As a result, AT-MEVs from these obese animals strongly reduced insulin-stimulated glucose uptake in SkM compared to AT-MEVs from lean animals. In vivo, the intravenous injection of AT-MEVs from obese to lean animals (30 μg every 7 days) significantly decreased SkM insulin sensitivity, demonstrated by a lower insulin-stimulated glucose disposal rates after AT-MEVs injections. Conversely, lean AT-MEVs improved SkM insulin sensitivity. Finally, the authors of this study demonstrated the specific role of miR-155 in the biological function of AT-MEVs, which targeted PPARα in the recipient SkM and consequently decreased GLUT4 and the insulin-induced phosphorylation of AKT [53].

### 3.4. Conclusion 1

- This work highlights the important role of miRNAs conveyed by EVs released by the different cell types that compose adipose tissue. It is likely that these miRNAs have a synergic role and that it is their combined action that contributes to the deterioration of muscle tissue in obesity. It would now be interesting to determine how the other constituents of EVs (lipids, proteins, and other ncRNAs) also contribute to the effects of these various EVs. Indeed, it is presently not known how obesity impacts the lipid composition of these EVs, which is an important parameter for EV incorporation into target cells, and which might also participate in the transfer of deleterious lipids between adipose tissue and muscle cells.
- It also appears that, within adipose tissue, EVs from mesenchymal stem cells have an important function in the maintenance and regeneration of muscle tissue, i.e., a protective effect on myocardium, the modulation of the microenvironment of smooth muscle cells, and the stimulation of SkM muscle regeneration. In the context of obesity, it seems clear that EVs from the adipocytes and macrophages of adipose tissue participate in alterations of SkM metabolic functions, e.g., the alteration of cardiac and smooth muscle cell contraction, the alteration of skeletal muscle and cardiac cell glucose uptake, and energy metabolism. On the other hand, the consequences of obesity on communication between mesenchymal stem cells and muscles through the EV route are not known. It would now be interesting to determine if mesenchymal stem cell-EVs keep their beneficial potential on muscle cells during obesity, if we want to consider stem cell-EVs as a mode of therapy to restore muscle mass.

## 4. Biological Action of Skeletal Muscle-Released EVs on Adipose Tissue Homeostasis

Until now, the role of muscle-released EVs on adipose tissue homeostasis has been little studied and has mainly been focused on the skeletal muscle EVs.

### 4.1. Muscle-Released EVs Healthy Context

There are around 600 muscles in the human body, which are divided into skeletal, smooth, and cardiac muscles. Inside muscular tissue, muscle cell-released EVs can act in a paracrine manner either on skeletal muscle cells [12,54] or on patrolling immune cells [55]. Surprisingly few experiments have been performed to determine whether muscle-released EVs can in turn also modulate adipose tissue homeostasis. There are few data focused on skeletal-muscle-released-EVs (SkM-EVs). In vitro, experiments have shown that EVs released ex vivo from myofibers [56], or released into the conditioned medium of SkM cells [45], can be rapidly incorporated into 3T3 adipocytes. In addition, it has been demonstrated that miRNAs contained in SkM-EVs are transferred into adipocytes, modulating gene expressions, i.e., the myomiR miR-133a is highly expressed in myofiber-derived EVs, downregulating its target proteins, Smarcd1 and Runx2, in the recipient adipocytes [56]. These data provide the first evidence that SkM-EVs can signal to nearby adipocytes in a paracrine manner. Interestingly, Runx2 downregulation in mesenchymal stem cells induces the expression of genes from the insulin pathways [57]. Therefore, it would be interesting to study the consequences on insulin-signaling and energy metabolism in adipocytes treated with SkM-EV-enriched miR-133a [56] to determine whether this miRNA can modulate lipogenesis and, consequently, adipogenesis [58]. Indeed, recent data have demonstrated that, in a healthy context, SkM-EVs prevent lipid accumulation in 3T3-L1 adipocytes [21] and inhibit fibro/adipogenic progenitors (i.e., tissue-resident mesenchymal stromal cells) from differentiating into adipocytes [59]. In addition, SkM-EVs released from hypertrophic SkM (i.e., hypertrophy induced by electric pulse stimulation or resistance exercise training) regulate catecholamine sensitivity and induce the release of glycerol from adipocytes compared to treatment with SkM-EVs from normal myotubes [59]. Taken together, these data demonstrate that SkM-EVs can control adipose tissue expansion locally and adjust the release of metabolites from the adipose tissue for proper SkM metabolism.

### 4.2. Muscle-Released EVs in Obesity

Pathological situations leading to muscle atrophy or impaired metabolism in obese or diabetic subjects affect the release, composition, and biological functions of SkM-EV [7]. Recently, one study demonstrated that atrophic insulin-resistant SkM from ob/ob mice release less EVs (OB-EVs) than SkM from WT mice (WT-EVs) [21]. OB-EVs were strongly enriched in cholesterol and in proteins from lipid metabolism. Very interestingly, this study showed that OB-EVs induce CD36, CIDEC, FABP4, and lipid storage in adipocytes, compared to WT-EVs. Therefore, compared to a ‘healthy’ situation [21,59], the state of obesity modifies the message released from SkM-EVs in favor of adipose tissue lipid storage and, thus, might participate in intramuscular adipose tissue expansion. Similarly, EVs released from vascular smooth muscle cells might participate in the control of perivascular adipocyte homeostasis. Indeed, vascular smooth muscle cells can capture and concentrate fetuin-A from blood and export it into various subpopulations of the EVs released into the microenvironment [60]. In parallel, one study demonstrated that fetuin-A inhibited adiponectin expression and induces insulin resistance in adipocytes [61], acting as a chemoattractant for macrophage migration, and that it could polarize M2 macrophages into proinflammatory M1 macrophages in adipose tissue [62]. Based on these data, it would therefore be interesting to study whether fetuin-A, conveyed by EVs and released from vascular smooth muscle cells, could participate locally in the dysfunction of adipose tissue during obesity, altering insulin sensitivity in adipocytes and favoring inflammation.

### 4.3. Muscle-Released EVs and Development of Adipose Tissue

Given the demonstration that SkM-EVs can modulate adipocyte metabolism and differentiation through the control of lipid storage, it is tempting to speculate that SkM-EVs may participate in the development of SkM and adipose tissue during embryogenesis. Indeed, during the development of vertebrates, muscles appear before the development of adipose tissue [63], and as muscle cells and adipocytes are both differentiated from mesenchymal cells, it is likely that the initial commitment of mesenchymal progenitors to the adipocyte lineage is put on hold until the muscle is fully developed and needs fuel from the adipose tissue. In a normal context, or during muscle regeneration, SkM-EVs act as anti-adipogenic signals, inhibiting the differentiation of fibro/adipogenic progenitors into adipocytes [56]. In addition, SkM-EVs collected during myotube differentiation can be internalized into adipose-tissue stem cells, inducing their fusion with neighboring cells to produce myotube-like cells expressing myosin heavy chain and desmin [64]. These data suggest that when muscle undergoes regeneration, SkM releases biologically active EVs to hijacks stem cells from adipose tissue in order to rebuild muscle and restore muscle mass. Even if regeneration is not development, it is likely that muscle cells release EVs during development, and therefore, it would be interesting to determine the role of SkM-EVs during the development of SkM and adipose tissue.

### 4.4. Conclusion 2

- Until now, the role of muscle EVs on adipose tissue homeostasis has been little studied. It was already known that, during muscle contractions, muscles secrete myokines which, by mobilizing fat from adipose tissue, contribute to the health effect of physical activity. With the studies mentioned above, it appears that skeletal muscle uses the EV pathway to control its homeostasis at the expense of the development of adipose tissue. This situation might start early, during vertebrate development. The control of adipose tissue expansion by muscle cell EVs is a new concept that needs further studies to determine which components of muscle EVs participate to this crosstalk.
- Obesity is often studied as a disease of adipose tissue (AT), and until now, the studies on crosstalk between this tissue and muscles have been focused on the role of AT-EVs in the alteration of muscle homeostasis. The few works described above indicate that, in addition to myokines, muscle EVs might participate quite early in the progressive dysfunction of the adipose tissue associated with obesity. Indeed, muscle insulin resistance appears before insulin resistance in other insulin-sensitive tissues during obesity-induced type 2 diabetes [65], suggesting that EVs from insulin-resistant muscles, by inducing the storage of lipids in adipose tissue, could induce this dysfunction. This hypothesis needs to be validated with in vivo experiments and for each type of muscle.

## 5. Perspectives

During development and throughout the life of an individual, muscle and adipose tissue control their respective mass and homeostasis by exchanging multiple signals, and recent data from the literature now show that extracellular vesicles are important partners in these exchanges. However, we are far from understanding the mechanisms of recognition and action of these EVs, and until now, only a few miRNAs have been identified. However, as EVs are lipid-derived nanovesicles, it is likely that their lipid composition, together with their protein content, plays an important role in their biological action, particularly in a context of obesity. Moreover, in the dialog between muscle and adipose tissue, the function of muscle-released EVs have been taken into account little. As highlighted in this review, recent work performed in vitro clearly indicates that muscle-released EVs control lipogenesis in adipose tissue. It will therefore be important to perform in vivo studies in humans, in normal situations, to validate these data. In addition, it will be also interesting to study whether sedentary lifestyles vs. physical activity [66], obesity, and aging alter muscle-EV release and composition in the same manner, and, consequently, how these affect the dialog between muscle and adipose tissue. Until now, only SkM-EVs have been taken into account in these dialogs. What about EVs released from the different types of muscles? Do they also modulate adipocyte lipid homeostasis in the same manner in different types of adipose tissue? Finally, it is not known whether the muscle–adipose tissue dialogue is ‘balanced’. A recent article indicates that ex vivo explants of SkM tissue from wild-type mice secrete more EVs than adipose tissue per unit of mass, and that SkM contractions do not modify this ratio [67]. Other studies indicate that the majority of blood extracellular vesicles might come from adipose tissue in the context of obesity in animal models [24,68]. These different studies are probably right, but time-course experiments to analyze the release and composition of muscle- and adipose tissue-EVs during the development of obesity, in the same subjects, could help to understand their respective contributions to the pathology.

## Data Availability

Not applicable.
